# Socio-demographic determinants associated with ownership and use of long lasting insecticide treated nets among pregnant women in the Wa Municipality of Ghana

**DOI:** 10.11604/pamj.2019.33.81.16245

**Published:** 2019-06-04

**Authors:** Ernest Darko, John Tetteh, Martin Amogre Ayanore, Ishmael Damoah-Aferi

**Affiliations:** 1 Department of Epidemiology and Biostatistics, School of Public Health, University of Health and Allied Sciences, Hohoe, Ghana; 2 Wa Municipal Health Administration, Ghana; 3 Department of Family and Community Health, School of Public Health, University of Health and Allied Sciences, Hohoe, Ghana; 4 Centre for Health Policy Advocacy, Innovation & Research in Africa (CHPAIR-Africa), Ghana; 5 Eastern Regional Pharmacy Council, Koforidua, Ghana

**Keywords:** Pregnant women, antenatal care, insecticide treated nets, long lasting insecticide nets, malaria, national malaria control program, Wa Municipality

## Abstract

**Introduction:**

An estimated 125 million pregnancies around the world are at risk of malaria infection every year. Insecticide Treated Bed Nets is a form of personal protection that has reportedly been shown to reduce severe disease and mortality due to malaria in endemic regions. This study investigated ownership and utilization of Long Lasting Insecticide Nets among pregnant women attending antenatal clinics in Wa Municipality of Ghana.

**Methods:**

A cross-sectional study design was adopted to collect data among 394 pregnant women in six antenatal clinics. A two stage sampling technique was adopted and the data collection tool used was a semi-structured questionnaire. Descriptive and inferential statistics involving logistic regression were performed using Stata 14.

**Results:**

More (33.3%) of the pregnant women were aged between 25-29 years with no formal education (29.9%) whiles most (69.6%) of the pregnant women were in Islam religion. About 95.9% have heard about Long Lasting Insecticide Nets and its benefits. Intuitively, ownership of Long Lasting Insecticide Nets was 82.2% with 69.3% utilization of Long Lasting Insecticide Nets. Pregnant women aged 30-34 and 35 years and above were significant predictors, however, less likely to own Long Lasting Insecticide Nets compared to 15-19 years [AOR(95%CI)=0.29(0.10-0.87) and 0.08(0.01-0.72) respectively] whiles pregnant women aged 35 years and above were significantly less likely to utilize Long Lasting Insecticide Nets compared to 15-19 years [OR(95%CI)=0.12(0.03-0.48)].

**Conclusion:**

The study found utilization of Long Lasting Insecticide Nets among pregnant in the Wa Municipality low as compared to the National Malaria Control Program target in Ghana although Long Lasting Insecticide Nets ownership was high. The study recommends that Public Health Nurses and Disease Control Officers should intensify sensitization on the importance and misconception of the use of Long Lasting Insecticide Nets during outreach clinics.

## Introduction

Globally, malaria is a public health problem in the world that threatens the lives of 3.2 billion people and leads to over one million deaths annually [1]. At least 300 million acute cases of malaria occur each year worldwide with about 90% of malaria deaths occurring in Africa [1, 2]. In sub-Saharan Africa (SSA), 80-90% of the world's malaria cases occur of which approximately 19-24 million women are at risk of malaria and its adverse consequences during pregnancy [3]. The use of Insecticide Treated Nets (ITNs) is one of the effective intervention strategy for the prevention of malaria in endemic areas [3, 4]. Awareness-use nexus of bed net use and the outcomes for malaria are reported in some studies in Africa. In Malawi for instance awareness of bed net was as high as 70%, while Long Lasting Insecticide Nets (LLIN) use was as low as 3% [5]. Furthermore, in Nigeria out of the 148 women who owned ITNs, 31.6% utilized ITNs among the 335 women studied [6]. Also, in Ethiopia, from the total households surveyed, 89.94% (456) own at least one LLIN in a household whiles 85.1% (388) had utilized LLIN the night before the survey [7].

In community-wide trials in several African countries bed nets have been shown to reduce child mortality by about 20% as well as reducing malaria incidence by about 50% [8]. Long lasting insecticide nets is a cost-effective measure adopted by the World Health Organization (WHO) aimed at reducing the incidence of malaria in endemic countries. The bed nets are treated with chemicals (parathyroid) to prevent mosquitoes from biting people whilst they sleep under the net. Depending on the type of chemical used, some remain potent for six months and others for years. This method has been tried and found to be effective in reducing the incidence of malaria in endemic countries including Ghana [8]. Malaria is the number one cause of morbidity accounting for 40.2% of all outpatient illnesses in Ghana [9]. ITNs is a form of personal protection that has reportedly been shown to reduce severe disease and mortality due to malaria in endemic regions. In Ghana, various types of ITNs have been on the market. They include the LLINs that require re-treatment only after about four years or twenty standard washes and the standard insecticide-treated nets that need to be re-treated every six months or after three washes [10]. This strategy is aimed at preventing mosquito contact, can aid in reducing infective bite and malaria transmission [11]. Malaria is hyper endemic in Ghana and among pregnant women, it accounts for 17.6% of outpatient department (OPD) attendance, 13.7% of admissions and 3.4% of maternal deaths [12]. A number of studies have investigated the awareness and utilization of mosquito bed net among pregnant women in Ghana. It was identified in Brong Ahafo, region, Ghana that, 96% of pregnant women were aware of the ITN and how it is used to prevent malaria [13]. Awareness about bed nets does not automatically translate into better utilization of LLIN especially in Ghana where the Ghana Demographic Health Survey report identified that, only 43% of pregnant women sleep under LLIN [14].

The attempt to control malaria in Ghana aimed at reducing malaria disease burden till it is of no public health significance began in 1950s [14]. In an effort to make mosquito nets more affordable, the government of Ghana has since 2002 waived taxes on the importation of nets into the country [14]. Development partners have also contributed by supplying some LLIN for distribution at subsidized costs to pregnant women and children under five in disadvantage areas. It has been the desire that the introduction of LLIN will help bring malaria under control. The LLIN has other advantages of controlling and prevention of yellow fever, lymphatic filariasis since these diseases are also transmitted by mosquitoes and also nuisances from other insects like houseflies, bed bugs and cockroaches [14]. Although Ghana has made significant progress over the last few years, much effort is still required to extend individual and household ownership coverage and use of ITNs. There are still 48.9% of households without ITN coverage and 57% of pregnant women not using ITN [14]. Given that a high proportion of pregnant women do not use ITN in Ghana as reported by Ghana Statistical Service, Ghana Health Service & ICF [14] is of public health concern. This study investigated socio-demographic determinants and their associations regarding the ownership and use of LLIN among pregnant women in Wa Municipality of Ghana.

## Methods

**Study site description:** the study was conducted in the Wa Municipality of Ghana. The Wa Municipality is one of the nine districts in the upper west region of Ghana. The study setting is located in the northern savannah part of the Ghana between Latitudes 8^o^ 30" - 10^o^ N and Longitude 0^o^ 30" - 2^o^ 30" W. Under the decentralization system in Ghana, the municipality is divided into 6 sub-municipals and 13 functional Community Health Planning and Services (CHPS) zones. CHPS zones are the lowest point of delivery for health care in Ghana. The population of Wa Municipal was estimated at 107,214 from the 2010 population and housing census [15].

**Study population:** the study population included pregnant women attending Antenatal Care in six sub municipals (Bamahu, Busa, Charia, Charingu, Kambali and Wa Central) in the Wa Municipality.

**Exclusion and inclusion criteria:** the study includes pregnant women attending Antenatal Clinic (ANC) in the Wa Municipality. Pregnant women who were not attending any ANC units where the data collection took place as well as pregnant women living outside the municipality were excluded from the study.

**Study design:** the study was a descriptive cross-sectional study design.

**Sample size determination:** the sample size included in the study was 394. This was calculated under the formula and procedure below.

$$n=\frac{z^{2}–pq}{d^{2}}$$

Where n = sample size required, Z = Z score, p = Proportion population, q = 1-p and d = margin of error. Z= 1.96 at 95% confidence level, p = 0.47, q = 1-0.47 = 0.53 and d = 0.05

$$n=\frac{1.96^{2}–0.47(0.53)}{0.05^{2}}$$

n = 382.77 = 383. Adding 3% non-respondent rate, n = (383 x 0.03) + 383 = 394

**Sampling method:** a two staged-sampling technique was adopted in the study. Simple random sampling technique was adopted to sample a Health Center each from Bamahu, Busa, Charia, Charingu, Kambali and Wa Central as the study zones. Proportionate sampling was then used to determine participants needed from each zone. Simple random sampling was finally adopted in each zone to recruit pregnant women for the study.

**Data collection procedure:** the data collection tool used was a semi-structured questionnaire. Fifteen (15) participants were used for pre-testing of the questionnaire at Wa urban health centre prior to final data collection to validate and ensure the quality of data. The languages that were used to administer the questionnaire were English, Waale and Dagaale.

**Data analysis:** data collected were entered into Epi Data version 3.1 and further exported to Stata 14 for data cleaning and analysis. Microsoft Excel version 2016 spreadsheet was used for charting. Descriptive and inferential statistics were performed. Descriptive involved cross-tabulations and also performing chi-square test to determine the associations between the dependent variable and independent variables. Inferential analysis involved binary logistic regression to assess predictors of ownership and utilization of LLIN among pregnant women in Wa municipality. A significant value was set at p-value < 0.05.

**Ethical consideration:** ethical approval was sought from the Ghana Health Service Ethics Review Committee with protocol ID: GHS-ERC/10/16 and permission was also obtained from the Wa District Health Directorate. Confidentiality was strictly observed.

## Results

**Figure 1:**
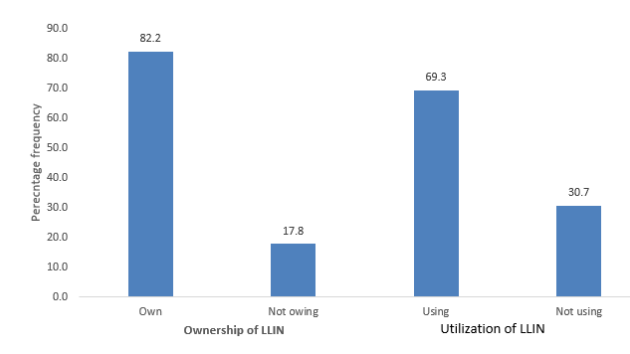
Ownership and utilization of LLIN among pregnant women

A total of 394 pregnant women took part in the study. More pregnant women 131 (33.3%) were aged between 25-29 years. Among the educational status, higher proportion 118 (30.0%) of the respondents had no formal education. Traders were more involved 129 (32.7%) whiles the least percentage among occupational status were civil servants were (19.8%). Moreover, Muslims pregnant women 268 (68.0%) were more involved in the study. Similarly, most of the pregnant women 358 (90.9%) were married (Table 1). Ownership and utilization of LLIN indicates that, majority of the pregnant women own LLIN in the Wa Municipality (82.2%) whiles the remaining 17.8% are not owing. However, 69.3% of the pregnant women utilized LLIN (Figure 1).

**Table 1:** Demographic characteristics of respondent

Variable	Frequency (N = 394)	Percentage (%)
**Age**		
15-19	37	9.4
20-24	97	24.6
25-29	131	33.3
30-34	93	23.6
35+	36	9.1
**Educational Status**		
Primary	64	16.2
Middle/JSS	85	21.6
Secondary	46	11.7
Tertiary	81	20.6
None	118	30.0
**Occupation**		
Civil servant	78	19.8
Trader	129	32.7
Farmer	55	14.0
Housewife	51	12.9
Seamstress/Hairdresser	81	20.6
**Marital Status**		
Married	358	90.9
Single	30	7.6
Widowed	6	1.5
**Religion**		
Christianity	126	32.0
Islam	268	68.0

**Associations between LLIN ownership and socio-demographic characteristics:** demographic variables were assessed to determine its association to ownership of LLIN among respondents. Age, educational status and occupation were statistically significant to ownership of LLIN (χ^2^(p-value) = 15.6(0.004), 11.69(0.019) and 10.33(0.035) respectively). Pregnant women aged 30-34 years and 35 years and above were 71% and 92% less likely to own LLIN as compared to age group 15-19 years respectively and were statistically significant (AOR=0.29, 95% CI 0.10-0.87; AOR=0.08. 95% CI 0.10-0.72 respectively). Moreover, pregnant women aged 20-24 years and 25-29 years were 39% and 53% less likely to own LLIN as compared to age group 15-19 years respectively but were not statistically significant (AOR=0.61, 95% CI=0.23-1.59 and AOR=0.47, 95%CI= 0.17-1.27 respectively). Regarding educational status, pregnant women who had attained tertiary level were 6.74 times more likely to own LLIN as compared to pregnant women with no formal education (AOR=6.74, 95% CI= 1.62-27.95). In terms of occupational with statistical significance, traders were 3.14 times more likely to own LLIN as compared to pregnant women in the civil sector (AOR=3.14, 95% CI=0.87-11.20) (Table 2).

**Table 2:** Associations and predictors of LLIN ownership with background characteristics

Attribute	Ownership of LLIN		Pearson Chi Square		OR (95% CI)	AOR (95% CI)
	Own	Not Own	χ^2^	P-value		
**Age**						
15-19	25(7.7)	11(15.7)	15.6	0.004**	**Ref**	**Ref**
20-24	75(23.2)	22(31.4)			0.67(0.28-1.57)0.052*	0.61(0.23-1.59)0.316
25-29	107(33.2)	25(35.7)			0.53(0.23-1.22)0.013**	0.47(0.17-1.27)0.135
30-34	82(25.3)	11(15.7)			0.30(.012-0.79)0.014**	0.29(0.10-0.87)0.027*
35+	35(10.8)	1(1.4)			0.65(0.01-0.54)0.011**	0.08(0.01-0.72)0.024*
**Education**						
Primary	58(17.9)	7(10.0)	11.69	0.019*	**Ref**	**Ref**
Middle/JHS	66(20.4)	19(27.1)			2.39(0.94-6.08)0.069	1.94(0.73-5.18)0.181
Secondary	33(10.2)	13(18.6)			3.26(1.18-8.99)0.022*	2.89(0.97-8.63)0.058
Tertiary	63(19.4)	18(25.7)			2.37(0.92-6.08)0.073	6.74(1.62-27.95)0.009**
None	104(32.1)	13(18.6)			1.04(0.39-2.75)0.944	1.45(0.51-4.08)0.486
**Occupation**						
Civil servant	63(19.4)	13(18.6)	10.33	0.035*	**Ref**	**Ref**
Trader	111(34.3)	24(34.3)			1.05(0.49-2.20) 0.002	3.14(0.87-11.20)0.046*
Farmer	50(15.4)	3(4.3)			0.29(0.79-1.08)0.064	1.09(0.19-6.02)0.922
House wife	36(11.1)	14(20.0)			1.89(0.79-4.45)0.148	3.61(0.94-13.86)0.062
Seamstress	64(19.8)	16(22.9)			1.21(0.54-2.72)0.643	3.63(0.91-14.36)0.066
**Marital Status**						
Married	286(88.3)	64(91.4)	0.15	0.699	**Ref**	
Single	32(9.9)	6(8.6)			0.84(0.34-2.09)0.704	-
Widowed	6(1.9)	0(0.0)			1	
**Religion**						
Christianity	101(31.2)	18(25.7)	0.83	0.361	**Ref**	
Islam	223(68.8)	52(74.3)			1.31(0.73-2.35)0.368	-

* P < 0.05

** p < 0.01

***p < 0.001

**Associations between LLIN utilization and background characteristics:** assessment of the association between demographic variables and utilization of LLIN showed that only age was statistically significant to utilization of LLIN (χ^2^=13.39, p=0.009). Inferential statistics was performed on the age groupings to determine the strength on the utilization rate. It was found that, 35 years and above have a strong influence on utilization rate but lesser as compared to age group within 15-19 years (OR=0.12, 95% CI=0.03-0.48). Age group 35 years and above were 88% less likely to utilize LLIN as compared to age group 15-19 years (Table 3).

**Table 3:** Associations between LLIN utilization and background characteristics

Attribute	Utilization of LLIN		Pearson Chi Square		OR (95% CI)
	Using	Not Using	χ^2^	P-value	
**Age**					
15-19	19(6.9)	17(14.1)	13.39	0.009**	**Ref**
20-24	62(22.7)	35(28.3)			0.68(0.29-1.64)0.392
25-29	96(35.2)	36(29.8)			0.45(0.19-1.05)0.065
30-34	65(23.8)	28(23.1)			0.48(0.19-1.15)0.100
35+	31(11.4)	5(4.1)			0.12(0.03-0.48)0.003**
**Education**					
Primary	47(17.2)	18(14.8)	4.45	0.348	**Ref**
Middle/JHS	58(21.3)	27(22.3)			1.63(0.73-3.64)0.238
Secondary	31(11.4)	15(12.4)			1.69(0.65-4.39)0.277
Tertiary	50(18.3)	31(25.7)			1.93(0.86-4.37)0.122
None	87(31.9)	30(24.7)			1.11(0.51-2.42)0.792
**Occupation**					
Civil servant	50(18.32)	26(21.49)	2.04	0.729	**Ref**
Trader	97(35.5)	38(31.4)			0.86(0.44-1.70)0.670
Farmer	38(13.9)	15(12.4)			0.71(0.29-1.75)0.459
House wife	33(12.1)	17(14.1)			1.31(0.58-2.98)0.520
Seamstress	55(20.2)	25(20.7)			1.04(0.50-2.15)0.915
**Marital Status**					
Married	242(88.6)	108(89.3)	0.31	0.857	**Ref**
Single	27(9.9)	11(9.09)			1.25(0.53-2.41)0.761
Widowed	4(1.5)	2(1.7)			1.57(0.26-9.55)0.625
**Religion**					
Christianity	83(30.4)	36(29.8)	1.79	0.18	**Ref**
Islam	190(69.6)	85(70.3)			1.43(0.84-2.45)0.187

*P < 0.05

** p < 0.01

***p < 0.001

## Discussion

Malaria is a major public health problem in the world that threatens the lives of 3.2 billion people globally and leads to over one million deaths annually. This study sought to investigate the utilization of LLIN among pregnant women in Wa Municipality. The findings of the current study provide significant information on pregnant women ownership and utilization of LLIN.

**Ownership of LLIN by pregnant women:** the study revealed that ownership of LLIN among respondents was 324 (82.2%). Although the national target of National Malaria Control Program (NMCP) for ownership of LLIN (100%) was not achieved, it was encouraging. The high ownership of LLIN among the pregnant women in Wa Municipality may be due to the free distribution of LLIN in Ghana since most LLIN are acquired for free [14]. Moreover, this could be attributed to, supplying of LLIN to pregnant women during antenatal visits. Respondents who had no formal education in this study were found to own more LLIN 104 (32.1). The study found out that age, educational status, and occupation were found as statistically significant predictor of pregnant women ownership of LLIN in this study. Pregnant women aged 30-34 years showed the highest level of association regarding ownership of LLIN in the study, followed by age group 35 years and above. This study showed that, pregnant women aged 30-34 and 35 years and above were 71% and 92% respectively less likely to own LLIN. Age was found as a predictor of ownership of LLIN in a research conducted in Mirab Abaya district, Southern Ethiopia. Due to differences in age classification to that of Tassew, Hopkins, & Deressa, (2017) [7] conducted in Ethiopia, they found out that, the age of respondents between 26 and 40 and 41-60 were 3.3-3.4 times more likely to own LLINs compared to those respondents whose age was either under 26 or over 60 years. In terms of educational status, pregnant women with tertiary educational level was found to have a high influence on ownership of LLIN. Pregnant women with tertiary educational level were 6.74 times more likely to own LLINs compared to those with no formal education. This could be explained that, education promotes empowerment and ensures development benefit through a continuous learning process, this makes the pregnant women to learn more about LLIN. Our finding goes in harmony with some literatures which found out that, respondents' level of education were significantly predictor with LLIN ownership [6, 7]. This current study further found out that, pregnant women who were traders showed the highest level of association regarding ownership of LLIN. The findings showed that, traders were 3.14 times more likely to own LLINs compared to civil servants. This finding could be explained that, traders work on their own and for that matter they can acquire LLIN whenever there is mass distribution of LLIN.

**LLIN utilization among pregnant women in the municipality:** utilization of LLIN in this study, was defined as pregnant women who reported to have slept under a LLIN the night before the survey were considered as the main users of LLINs [14]. In this study, a total of 273 (69.3%) pregnant women slept under LLIN the previous night before the survey whiles 121 (30.7%) did not. Even though more than half of the pregnant women (69.3%) utilize LLIN the previous night, it was not encouraging. Our findings showed much higher than a similar one conducted in northern Uganda which showed 20.9% of pregnant women slept under LLIN a night before the survey [16] and lower utilization (32% and 40.7%.) of pregnant women slept under ITN a night before the survey in Nigeria [6, 17]. Feeling hot to sleep under LLIN, not having LLIN and prefer using mosquito spray were the reasons preventing them from sleeping under LLIN. To confirm the utilization improvement (69.3%) by pregnant women in Wa Municipality, a similar research in Kasena - Nankana district in Ghana showed that out of 80% of women who own nets, about 70% of women used them frequently [18]. According to Deladem (2013), the use of LLIN was high among pregnant women of northern tribes as compare to other groups [19]. Moreover, this current study identified only age as significant predictor of pregnant women utilization of LLIN. The findings of Deladem (2013) again confirms that age is predictor of pregnant women utilization of LLIN [19].

## Conclusion

The study discovered that LLIN ownership was high. Although utilization was low as compared to the NMCP target, it has been improved. It is being understand that, owning LLIN facilitates its use but this study concludes that, owning LLIN does not necessarily make one to use. Pregnant women who did not use LLIN stated reasons such as embarrassments due to heat, lack of ownership of LLIN and preference of using mosquito spray as some of the barriers to LLIN utilization. Although the target of the NMCP 85% utilization of LLIN were not met, there has been an improvement on the ownership and utilization of LLIN by the findings of this study.

**Recommendations:** this study recommends that Public Health Nurses and Disease Control Officers should intensify sensitization on the importance and misconception of the use of LLIN during outreach clinics as well as local radio presentations.

### What is known about this topic

- 43% of pregnant women sleep under LLIN from the 2014 Ghana demographic health survey;
- 57% of pregnant women did not sleep under ITN from the 2014 Ghana demographic health survey;
- 51.1 % of households own ITN from the 2014 Ghana health demographic survey.

### What this study adds

- 69.3% of the pregnant women slept under LLIN the previous night before the survey;
- 30.7% of the pregnant women did not sleep under LLIN the previous night before the survey;
- 82.2% of pregnant women own LLIN.

## Competing interests

The authors declare no competing interests.
